# Trained immunity: induction of an inflammatory memory in disease

**DOI:** 10.1038/s41422-025-01171-y

**Published:** 2025-10-14

**Authors:** Titus Schlüter, Yuri van Elsas, Bram Priem, Athanasios Ziogas, Mihai G. Netea

**Affiliations:** 1 Department of Internal Medicine and Radboud Center for Infectious Diseases, Radboud University Medical Center, Nijmegen, The Netherlands; 2 Department of Cell Biology, Faculty of Science, Radboud Institute for Molecular Life Sciences, Radboud University, Nijmegen, The Netherlands; 3 Department for Immunology and Metabolism, Life and Medical Sciences Institute (LIMES), University of Bonn, Bonn, Germany

**Keywords:** Innate immunity, Tumour immunology, Mechanisms of disease, Epigenetics

## Abstract

The innate immune system adapts its behavior based on previous insults, mounting an enhanced response upon re-exposure. Hematopoietic progenitors in the bone marrow and peripheral innate immune cells can undergo epigenetic and metabolic reprogramming, establishing an innate immune memory known as trained immunity. The concept of trained immunity recently gained relevance in our understanding of how innate immunity is regulated in various diseases. This review explores the role of trained immunity in infections, autoimmune disease, cardiovascular disease, cancer, and neurodegenerative disease. We discuss how trained immunity can provide heterologous protection against infections, as it has been induced for decades by the Bacillus Calmette Guérin vaccine, how it can help counteract immunosuppression, and how it can be inappropriately induced leading to chronic inflammation. By understanding how trained immunity is involved in processes leading to health and disease, novel therapeutic strategies can be developed.

## Introduction

Our body is constantly surveilled by innate immune cells, ready to initiate host defense or repair responses. Upon recognizing molecular patterns associated with pathogens or damage, innate immune cells initiate inflammation with the release of inflammatory mediators. In contrast to specific recognition of antigens by adaptive immunity, innate immune cells evolved pattern recognition receptors (PRRs) to recognize a wide range of pathogen-associated molecular patterns (PAMPs), molecular microbial signatures, and damage-associated molecular patterns (DAMPs) released by injured or dying cells. Upon activation, innate immune cells employ effector functions for clearance of pathogens or cell debris, induce adaptive immune responses, and support the repair of the tissues. On the other hand, dysregulated innate immune responses can cause chronic inflammation, which underlies a multitude of non-communicable diseases. An appropriately regulated innate immune system is critical to fight off infections and maintain health. This makes modulation of innate immunity of high therapeutic interest.^1^

The memory of an organism allows it to adapt its behavior based on past experiences. While our primary experience of this is our own neurological memory, memory extends beyond this. In that way, memory is observed in many cells of our immune system. Vaccines as one of the most effective medical interventions target this memory.^2^ Typically, a vaccine elicits specific immune memory in lymphocytes. This adaptive immune memory is based on genomic rearrangement and hypermutation, generating antigen-specific T cell receptors and immunoglobulins.^2^ These receptors recognize previously experienced antigens, triggering specific memory immune responses. In addition to adaptive immunity, research in the last decade led to the discovery of memory characteristics of innate immune cells termed ‘trained immunity’. Indeed, many populations of immune and non-immune cells, and arguably all living organisms, including plants and bacteria, display some form of functional memory.^3–6^ Altered responses based on past experiences, the hallmarks of memory, are widely apparent in innate immunity across organisms.^7–9^ The discovery of this innate immune memory opens novel avenues for prophylactic and therapeutic intervention.^10^

Long-term memory as trained immunity can be maintained in short-lived innate immune cells. This is mediated at the level of hematopoietic stem and progenitor cells (HSPCs). These cells reside in the bone marrow and give rise to all blood cell lineages. Output and lineage differentiation of HSPCs are under constant regulation. This allows adaptations to physiological demands and insults, such as vaccines, infections and sterile inflammation. Commonly, these insults lead to the expansion of progenitor populations and an increased hematopoietic output biased towards myeloid and megakaryocytic lineages.^11,12^ In response to inflammatory triggers, HSPCs can store experienced information through epigenetic and metabolic reprogramming.^13^ This allows sustained adaptations in hematopoiesis past the initial insult.^14^ HSPCs pass these epigenetic programs on to their progeny. This results in an adapted behavior of the generated effector cells. Effectively, this makes the bone marrow a repository of innate immune memory, which can be recalled for adapted effector functions, a phenomenon termed central trained immunity.^9,14^ Meanwhile in peripheral tissues, self-maintaining innate immune cell lineages of fetal origin, such as lung alveolar macrophages or microglia in the brain, undergo their own reprogramming in the periphery. This process is termed peripheral trained immunity. Both central trained immunity and peripheral trained immunity shape the behavior of innate immune cells for up to one-two years duration.^9^

Trained immunity enables enhanced immune function in response to infections, improving pathogen clearance and resolution of inflammation. However, if inappropriately activated, trained immunity can be maladaptive and promote chronic and hyperinflammatory responses to sterile insults. In the context of severe inflammatory events innate immune memory can also lead to immunosuppression, increasing the susceptibility to infection. Efforts are made to understand trained immunity in the context of diseases, how it connects pathologies and fuels co-morbidities, and can be leveraged for therapy.

## Mechanisms for induction of trained immunity

Trained innate immune cells display memory characteristics and adapt their effector functions based on this memory. Depending on the biological context of an insult, trained immunity programs can lead to enhanced or suppressed effector functions. This can lead to improved pathogen clearance through phagocytosis, release of reactive oxygen species (ROS) and release of pro-inflammatory cytokines upon activation through PRRs, but also immunosuppressed phenotypes.^1,9^

**Fig. 1:**
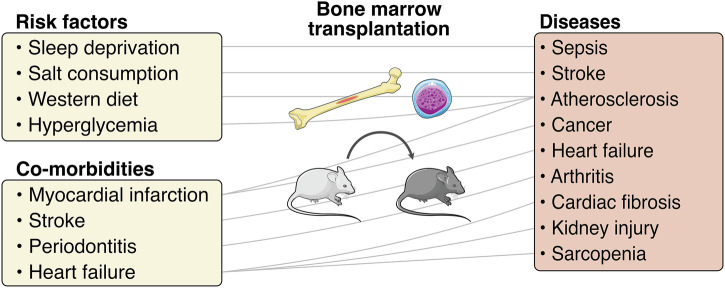
Bone marrow transplantation studies identify maladaptive trained immunity as a link between inflammatory co-morbidities. Bone marrow transplantation studies provide valuable evidence for central trained immunity being a causative link that aggravates disease models in mice. In the naïve recipient mice, only the transplanted HSPCs have been exposed to the training stimulus and are therefore the only population of cells responsible for observed phenotypes. This association of disease through bone marrow HSPCs was demonstrated for sleep deprivation and sepsis survival,^171^ high salt consumption and stroke,^172^ diet-induced inflammation and atherosclerosis,^20^ hyperglycemia and atherosclerosis,^17^ myocardial infarction and atherosclerosis,^19^ myocardial infarction and cancer,^173^ stroke and heart failure,^22^ periodontitis and arthritis,^18^ heart failure and cardiac fibrosis, heart failure and kidney injury, heart failure and sarcopenia.^21^

Trained immunity is induced by immunological signaling and metabolic reprograming mediated by hypoxia-inducible factor 1-α (HIF1-α) downstream of mammalian target of rapamycin (mTOR), directing a shift towards aerobic glycolysis and allowing accumulation of acetyl-coenzyme A and tricarboxylic acid cycle derived metabolites. These metabolites fuel the activity of histone modifying enzymes.^9^ Epigenetic reprogramming in trained immunity is mediated through deposition of H3K4me1, H3K4me3, H3K18la, and H3K27ac histone marks in promoter or enhancer regions of inflammatory response genes leading to permissive chromatin and facilitated expression.^9,15^ Trained immunity is observed in arguably all peripheral innate immune cells, including tissue-resident self-renewing macrophage populations and innate immune cells of hematopoietic origin, such as neutrophils, monocytes, macrophages, natural killer (NK) cells and innate lymphoid cells (ILCs). Trained immunity of short-lived cells is maintained through long-term epigenetic and functional reprogramming of their hematopoietic progenitors.^9^ Protective trained immunity effects are induced against infections by certain vaccines, while inappropriate induction of trained immunity by endogenous ligands can aggravate many pathological conditions including atherosclerosis, periodontitis, arthritis, or gout^16–24^ (Fig. 1).

## Inflammatory memory in disease

### Infectious disease

**Fig. 2:**
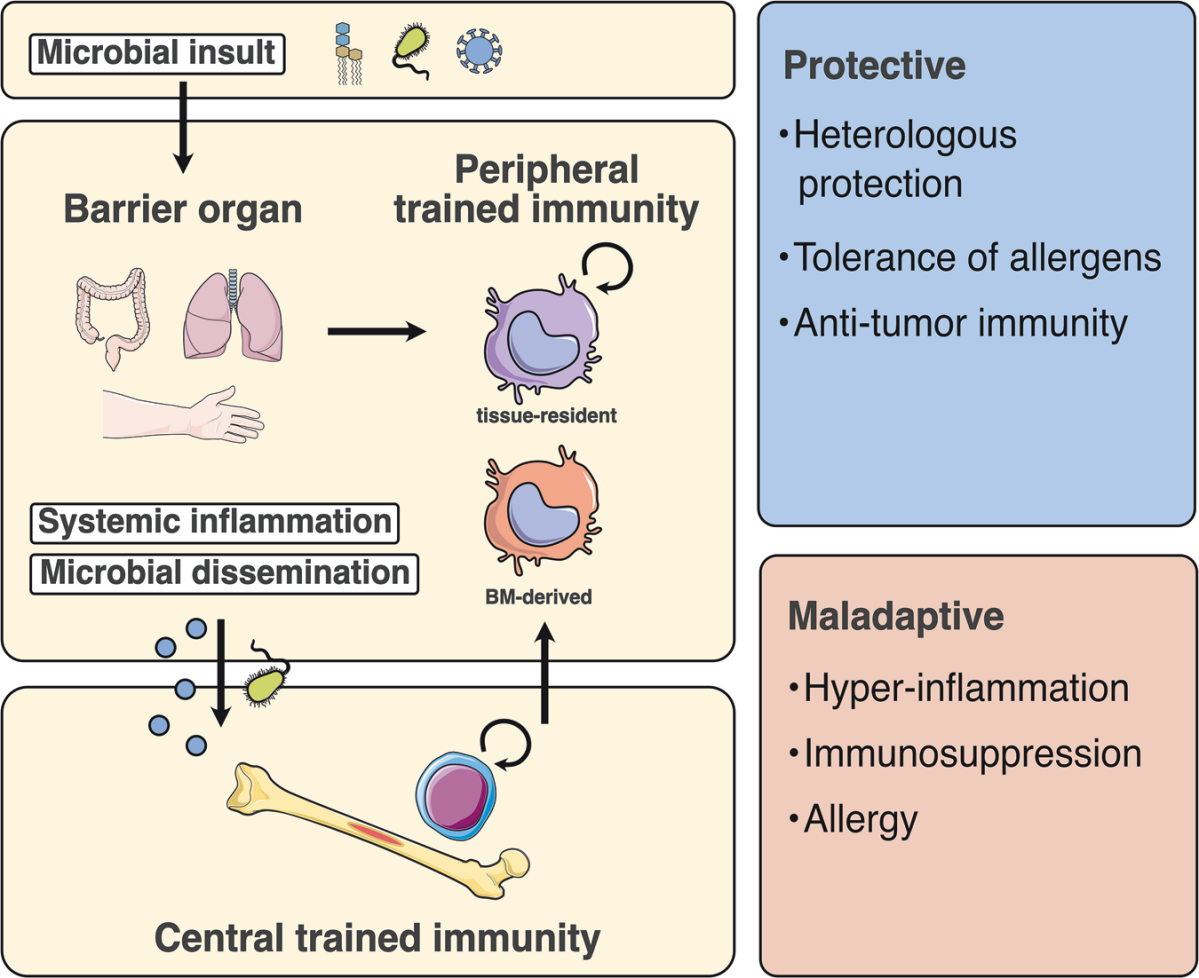
Central and peripheral reprogramming upon infection. A microbial insult is initially faced at the barrier organ of entry triggering activation and peripheral trained immunity of tissue-resident innate immune cells and local inflammatory signaling. Systemic inflammatory signals and microbial dissemination into the system can induce central trained immunity in the bone marrow. Trained innate immune cells egress from the bone marrow as effector cells representing central trained immunity in barrier organs. Induced in the appropriate contexts, trained immunity can protect health through heterologous protection against pathogens of similar biological context, enhanced tolerance of allergens and even promote antitumor immunity.^40,52,56,75^ In other contexts, induction of trained immunity can have adverse effects, leading to hyper-inflammation, increased susceptibility of infection and exacerbated allergy.^57,66,80^

Host defense against infectious diseases depends on both innate and adaptive host defense mechanisms. And both peripheral and central trained immunity are relevant. For instance, in the lung and respiratory tract, self-renewing tissue resident alveolar macrophages and bone marrow derived interstitial macrophages can be exposed to training inducing stimuli.^25^ Additionally, the tissue environment is known to imprint functional programs onto resident and infiltrating innate immune cells, regulating their phenotype and plasticity.^26^ In different contexts, the combination of these adaptations can have protective or adverse effects (Fig. 2).

The discovery of trained immunity is tightly linked to the live attenuated tuberculosis vaccine Bacillus Calmette Guérin (BCG) generated from *Mycobacterium bovis* more than a century ago. In children, BCG vaccination decreases all-cause mortality. Early on, experimental models demonstrated heterologous protective effects of BCG against *Listeria monocytogenes* and *Salmonella typhimurium* infections, as well as early tumor mouse models.^27,28^ Similar protective effects can be observed in mice challenged with innate immune triggers such as fungal cell-wall component β-glucan, the peptidoglycan fragment muramyl dipeptide, but also hemozoin accumulation through malaria infection.^29–32^ Protective effects were found to persist in severe combined immunodeficient (SCID) mice lacking functional T and B lymphocytes, suggesting protective memory to be independent of adaptive immunity.^33^

BCG vaccination and mycobacterial infections have been used as a tool to study trained immunity. This provided important insights into the mechanisms mediating training. BCG stimulates emergency hematopoiesis and induces trained immunity through intracellular receptor Nucleotide-binding oligomerization domain-containing protein 2 (NOD2), interleukin-1β (IL-1β) and interferon-γ (IFN-γ) signaling. Permissive histone marks and open chromatin regions facilitate transcription factor activity for myeloid differentiation and inflammation. Nuclear factor kappa light chain enhancer of activated B cells (NF-κB) and activator protein 1 (AP-1), as well as interferon responsive factors, such as signal transducer and activator of transcription 3 (STAT3) are involved in BCG training.^34–36^ Like BCG, the highly virulent *Mycobacterium tuberculosis* also colonizes the bone marrow. However, there it employs effector functions to suppress the induction of trained immunity and myeloid lineage output by manipulating iron metabolism in myeloid progenitors, leading to necroptosis.^37^ Trained immunity is induced in mice challenged with *Mycobacterium avium*, characterized by lasting transcriptional activation of HSPCs, which could be mimicked with a single dose of IFN-γ.^38^ Prolonged IFN-γ signaling upon chronic *M. avium* infection can deplete the HSPC pool by driving myeloid differentiation.^39^ *Μ. avium*-induced trained immunity, arguably directed against intracellular pathogens, could be transferred through bone marrow transplantation into naïve recipient mice and enhance protection against influenza infection.^38^ Mild protection against *M. avium* infection was also observed upon influenza-induced trained immunity.^38^ This suggests overlaps in trained immunity signatures with a T helper 1 type signature, directed against viruses and eventually tumors. In line with this, severe influenza infection induces trained immunity that promotes anti-tumor immune responses, protecting against primary lung metastasis in a mouse melanoma model.^40^ Adoptive transfer of alveolar macrophages in naïve mice was sufficient for inducing the beneficial effects, suggesting peripheral trained immunity to be the protective factor.^40^ In such adoptive transfer experiments, it is important to consider confounding effects caused by the replacement of lung macrophage populations with bone marrow-derived macrophages with higher reactivity.^41^ As the tissue environment has a strong regulatory function on resident innate immune cells, studies employing adoptive transfer of tissue resident innate immune cells are a useful tool to separate the effects of an altered tissue environment and peripheral trained immunity. Similarly, other studies employed adoptive transfer post adenoviral vaccination, tracing back protective effects upon *Streptococcus pneuomniae* infection to trained alveolar macrophages.^42^ In contrast, intranasal lipopolysaccharide (LPS) challenge displayed protective trained immunity effects upon *S. pneumoniae* infection in the same mouse, but lead to increased susceptibility upon alveolar macrophage transfer into naïve mice, suggesting a more prominent role of the tissue environment.^43^

Signatures of epigenetic memory are found in monocytes of individuals post severe acute respiratory syndrome coronavirus 2 (SARS-CoV-2) infection.^44^ Monocytes and hematopoietic progenitors from individuals that suffered severe coronavirus disease 2019 (COVID-19) maintained epigenetic trained immunity signatures for up to one year.^45^ Differences in chromatin accessibility suggest IL-6 signaling as the factor that initiates training.^45^ IL-6 receptor (IL-6R) blockade during COVID-19 attenuated the expansion of granulocyte-monocyte progenitors (GMPs).^45^ Open chromatin at binding sites of IL-6R target STAT3, and myelopoiesis-regulating CCAAT/enhancer-binding-protein (C/EBP) family transcription factors could contribute to the lasting increase in GMPs and hyperresponsive monocytes post severe COVID-19.^45^ In another study, mice that recovered from SARS-CoV-2 infection displayed peripheral trained immunity in lung alveolar macrophages, marked by permissive chromatin at interferon-stimulated gene loci.^46^ Peripheral trained immunity in alveolar macrophages was associated with enhanced cytokine responses and increased protection against influenza infection.^46^ Thus, a cleared SARS-CoV-2 infection can provide heterologous protection, but may also have maladaptive effects depending on COVID-19 severity.

In the context of a pandemic, trained immunity-inducing vaccines could be an important tool to bridge the development of specific vaccines. This is especially relevant for aged populations that are less responsive to vaccination while facing increased risk of severe infections.^47,48^ There is contrasting clinical evidence on whether BCG vaccination can still induce trained immunity for cross-protection in aged populations.^49–51^ While protecting against influenza, the BCG vaccine appears to offer limited heterologous protection against SARS-CoV-2 in both young and aged adults.^52,53^ However, BCG-vaccinated elderly display decreased inflammatory plasma proteins during COVID-19, suggesting a favorable immunoregulatory effect.^54^

Similarly, induction of trained immunity in alveolar macrophages through intranasal LPS protected mice from *S. pneumoniae*, but provided minimal protection against SARS-CoV-2 infection.^55^ Viral infections could be expected to provoke overlapping training signatures. However, different viral pathogens may lead to different secondary responses. For instance, infection of mice with murine herpesvirus leads to immune tolerance in a house dust mite (HDM) allergy model, whereas early life infection of mice with enterovirus 71 exacerbates inflammation upon HDM challenge.^56,57^ These studies suggest that we need to further study different trained immunity-inducing vaccines and the overlapping biological contexts of the pathogens they protect against.

Recent studies showcase epigenetic memory in peripheral cells other than macrophages to manage bacterial infection. Gut infection of mice with *Citrobacter rodentium*, a model for enteropathogenic *Escherichia coli*, was demonstrated to confer protection against secondary infections through induction of trained immunity in type 3 ILCs.^58^ Fibroblastic reticular cells in gut associated lymphoid organs support humoral responses against *C. rodentium* which is improved upon epigenetic reprogramming induced by mild dextran sulfate sodium (DSS) colitis.^59^ Urinary tract infection with uropathogenic *E. coli* was shown to induce epigenetic reprogramming in urothelial stem cells. This results in altered shedding and inflammatory responses upon infection of the urothelium.^60^

Commensal microbiota colonizing our barrier organs are a source of various metabolites and PAMPs with immunomodulatory roles. High abundance of commensal bacteria triggers innate and adaptive responses that enhance barrier organ immunity.^61^ Beneficial effects may not be limited to that. For instance, dietary *Lactiplantibacillus plantarum* IMB19 enhances sustained macrophage antitumor immune responses in mice.^62^ Intraperitoneal injection with commensal *Enterococcus faecalis* shows heterologous protection against infection through trained immunity.^63^ The mediating PAMP was identified as ribosomal protein S11 and showed effect as an adjuvant, enhancing protective effects of influenza vaccination.^63^ On the other side, non-communicable diseases, such as allergy, inflammatory bowel disease and multiple sclerosis are associated with dysbiosis and immune dysregulation.^64^

When an infection escalates into sepsis, innate immune responses can become life-threatening. Survivors of the acute phase of excessive inflammation can suffer compromised immune protection. Post-sepsis, patients are at increased risk of infection for up to one year post-discharge.^65,66^ Conceptually, trained immunity induced by β-glucan can reverse LPS-induced tolerance.^67^ Thus, trained immunity may have therapeutic use to counteract immunosuppression. Agents such as β-glucan are eventually not compatible with acute immunosuppressive treatment. Because of this, IL-4 emerges as a promising candidate. IL-4 inhibits acute inflammation through STAT6 while inducing trained immunity through the phosphoinositide 3 kinase (PI3K)/mTOR pathway.^68^ This was demonstrated effective using IL-4 anchored to lipid nanoparticles, targeting myeloid phagocytes and the bone marrow.^68^ Effective drug delivery is essential for therapeutic use of cytokines. While some recombinant cytokines are used for therapy, cytokines share some limitations regarding their pharmacological use, such as pleiotropic effects in many different cell types. Recombinant cytokines often have poor pharmacokinetics related to the small size of the proteins.^69^ Protein modifications and novel delivery systems are being investigated to improve these characteristics. This can enhance receptor, cell type and tissue specificity in conjunction with more favorable pharmacokinetics.^70,71^

A screen of small molecule libraries for induction of trained immunity identified glucocorticoids such as flunisolide and hydrocortisone as inducers of trained immunity, while suppressing acute inflammation.^72^ Chromatin accessibility analysis suggests different programs of epigenetic memory induced by glucocorticoids compared to β-glucan. As hydrocortisone is effective and widely applied for the treatment of community acquired pneumonia and septic shock, further studies are needed on how these trained immunity effects could protect against reinfection.^73,74^

Intriguingly, epidemiological studies find decreased incidence of cancer in sepsis survivors.^75^ At the same time, incidence of cardiovascular complications in sepsis and severe pneumonia survivors is increased.^76–79^ Notably, cardiovascular complications in other severe disease survivors are commonly observed.^77^ This suggests that both lasting immunosuppression and maladaptive trained immunity could cause complications post-sepsis.

In mouse models of sepsis, hallmarks of pro-inflammatory trained immunity can be observed in the bone marrow.^80^ This may need to be viewed separately from immunosuppression observed in tissues such as the lung.^81^ Sepsis is described to induce stressed phenotypes in granulocytes through innate immune memory. These reprogrammed granulocytes can contribute to immunosuppression as myeloid derived suppressor cells (MDSCs), but at the same time aggravate organ damage through excessive pro-inflammatory cytokine release in an event of secondary infection.^82,83^ Post-sepsis effects on cancer were examined in a mouse model of respiratory sepsis recapitulating the protective effects.^75^ This was linked to induction of trained immunity in tissue resident macrophages and hematopoietic progenitors, resulting in favorable alterations in tissue resident T cell compositions.^75^

Sepsis has a complex pathology with spatial and temporal factors impacting long-term effects of immunosuppression and trained immunity. In such complex pathophysiology, innate immune reprogramming may not be viewed as a singular event but a dynamic process. Innate immune cells and cells of the tissue environment likely face multiple bouts of different triggers consisting of bacterial PAMPs and/or endogenous inflammatory mediators, that can have differing effects on their reprogramming.^84^ Treatment of sepsis and associated complications using the concepts of trained immunity poses challenges of timing and tissue or cell type specificity.

### Autoimmune disease

Autoimmune diseases are characterized by a dysregulated response against self-antigens. Autoreactive T cells and autoantibodies produced by plasma B cells are main drivers of these responses. For many autoimmune diseases, the innate immune system has been recognized as a contributing factor and therapeutic target.^85,86^ Trained immunity may contribute to dysregulation of innate immune cells in autoimmune disease, both directly or indirectly by modulating lymphocyte function.^85,87^ The role of hematopoietic activity and trained immunity in the bone marrow and periphery have been studied for prevalent autoimmune diseases. This includes systemic lupus erythematosus (SLE), multiple sclerosis (MS) and its mouse model experimental autoimmune encephalitis (EAE), as well as rheumatoid arthritis (RA) and related mouse models.

SLE involves multiple organs and is marked by immune responses against nuclear self-antigens. In a mouse model of SLE, administration of β-glucan was demonstrated to exacerbate disease.^88^ Emergency myelopoiesis signatures can be observed in SLE.^89^ In a pristane-induced mouse model for SLE, trained immunity was demonstrated in epigenetically and metabolically reprogrammed hematopoietic stem cells (HSCs), giving rise to macrophages with enhanced inflammatory cytokine response and killing of *M. avium*.^90^ Upon bone marrow transplantation into naïve recipient mice, autoimmune-trained HSCs maintained epigenetic and functional trained immunity, while reverting metabolic alterations.^90^ Nuclear self-antigens such as apoptotic microparticles and neutrophil extracellular traps (NETs) isolated from the circulation of SLE patients can induce trained immunity in vitro.^91^ Trained immunity induced by nuclear antigens was linked to increased IL-6 responses in peripheral blood mononuclear cells (PBMCs) from non-flaring SLE patients.^91^

In the EAE mouse model, trained immunity through exposure to BCG or helminth secretory products was shown to attenuate disease.^92,93^ While therapeutic potential of inducers of trained immunity could be limited due to their potential to exacerbate extant neuroinflammation, innate immune responses underlie reparatory remyelination responses that can counteract demyelinating injury associated with MS. In aged mice, BCG vaccination was demonstrated to enhance microglial remyelination to the same extent as deleting microglial histone deacetylases 1 and 2.^94^ This suggest that in the context of MS, appropriate induction of trained immunity could be beneficial. Other studies demonstrate maladaptive training. Emergency hematopoiesis, instructed by autoreactive T cells in the bone marrow, was recently described as a driving factor in MS progression, displaying expansion of progenitors and myeloid skewing reminiscent of myelopoiesis influenced by central trained immunity in mice.^14,95^ Microglia resident in the central nervous system (CNS) are known to be able to switch to a primed state reminiscent of trained immunity.^96^ Microglia were demonstrated to be primed through ablation of a C3 convertase regulating protein, resulting in increased complement signaling.^97^ This microglial reprogramming aggravated pathology in EAE.^97^ Epigenetic memory was recently described in astrocytes, mediated by ATP-citrate lyase and histone acetyltransferase p300.^98^ Astrocyte memory phenotypes were more pronounced in EAE and contributed to pathology.^98^ In summary, while administration of BCG or helminth secretory products could be beneficial for treatment of MS, adverse effects reminiscent of maladaptive trained immunity cannot be excluded and should be monitored attentively.

In RA, evidence of trained immunity is mostly incriminating. In a genetic mouse model prone to phenotypes resembling RA, β-glucan can be employed to initiate the disease.^99^ Along these lines, β-glucan-induced trained immunity can drive the generation of osteoclasts, cells of the myeloid lineage mediating inflammatory bone erosion.^100^ Through this mechanism, β-glucan training was shown to increase inflammatory bone loss in mouse models of periodontitis and RA.^100^ The mouse model of collagen-antibody induced arthritis was shown to trigger heightened myelopoiesis responses.^18^ In the same study, bone marrow transplantation experiments between arthritis and periodontitis models demonstrated IL-1β-mediated maladaptive central trained immunity as an underlying factor of co-morbidity.^18^ Tissue resident immune cells can contribute to autoimmune disease through their own maladaptive trained immunity. LPS injections in mouse joints were shown to exacerbate inflammation upon local *Staphylococcus aureus* infection 21 days later.^101^ Tissue resident synovial macrophages displayed signatures of innate immune memory such as upregulated mTOR phosphorylation and H3K4me3 deposition.^101^ Synovial fibroblasts are found epigenetically and metabolically reprogrammed in inflammatory arthritis, reminiscent of trained immunity and contributing to tissue inflammation and erosion.^102^

These studies suggest a crosstalk between induction of trained immunity and susceptibility to autoimmune disease. While appropriate induction of trained immunity could be beneficial in MS, these studies mostly demonstrate trained immunity as a target of therapeutic intervention.

### Other autoinflammatory diseases

Evidence of ex vivo cytokine responses and epigenetic alterations in patient monocytes suggest a contribution of trained immunity to other autoinflammatory diseases including familial autoinflammatory diseases, gout, sarcoidosis, systemic sclerosis and inflammatory bowel disease.^103–105^

Hyper immunoglobulin D syndrome (HIDS) is a monogenic autoinflammatory disease caused by deficiency of the enzyme mevalonate kinase, an early enzyme in cholesterol synthesis. Mevalonate kinase deficiency leads to accumulation of the intermediate mevalonate.^106^ Mevalonate is involved in the induction of trained immunity in response to β-glucan.^107^ Mevalonate itself induces training through insulin-like growth factor 1 receptor (IGF1-R).^107^ This induced training may underlie the activated phenotype observed in HIDS monocytes regarding expression of mTOR- and glycolysis-related genes as well as ex vivo cytokine responses.^107^

Hyperuricemia is an initiating and crucial factor in the pathogenesis of gout. Hyperuricemia was demonstrated to prime monocytes for increased inflammatory cytokine responses.^24^ IL-1β release upon activation of the NOD-like receptor family pyrin domain containing 3 (NLRP3) inflammasome in response to monosodium urate crystals is involved in gout, which is a relevant inducer of pro-inflammatory trained immunity.^108^ Alterations in histone modifications are described in gout patient monocytes and monocytes primed with urate.^23,24^

Systemic sclerosis is an auto-inflammatory disease marked by extensive fibrosis in the skin, internal organs and vasculature. In a mouse model for systemic sclerosis, adoptive transfer of macrophages from low-dose LPS or BCG trained mice impacted disease severity.^109^ BCG training worsened and low-dose LPS ameliorated disease, suggesting that peripheral trained immunity impacts systemic sclerosis.^109^

Sarcoidosis is an auto-inflammatory granulomatous disease of unknown cause. Hypotheses about the etiology of sarcoidosis include microbial and environmental triggers as well as genetic factors. Indications of the hallmarks of trained immunity, mTOR signaling activity, metabolic reprogramming and epigenetic rewiring, are reported.^105,110–113^ Thus it is proposed that maladaptive trained immunity is involved in sarcoidosis pathogenesis.^105^ Importantly, trained immunity signatures are found not only in granulomatous tissues, but also in circulating monocytes.^114^ A rare set of sarcoidosis patients undergoing lung transplantation suffered recurrence of granulomas. Immune cells in recurrent granulomas were found to be recipient-derived.^115^ Sarcoidosis patient monocytes differentiated ex vivo maintain aberrations in lipid metabolism, highlighting the internal dysregulation of these cells even in absence of eventual in vivo stimuli.^110^

### Atherosclerotic cardiovascular disease

Atherosclerotic plaques can accumulate in medium-sized and large arteries over the lifetime, ultimately leading to atherosclerotic cardiovascular disease (ASCVD). Current therapies are aimed at ameliorating hyperlipidemia and hypertension but the risk reduction of recurring ASCVD remains low.^116^ The CANTOS and LoDoCo trials previously revealed that atherosclerosis is an inflammatory disease, suggesting that patients may benefit from immunotherapies tailored to ASCVD.^117,118^ Furthermore, though hyperlipidemia is not the sole driver of atherosclerotic progression, it is a vital inducer of inflammation in ASCVD.^16^

Recent work has demonstrated how intermittent periods of high-fat and conventional chow diet can induce a potent trained immunity response in atherosclerosis-prone *Ldlr*^*−/−*^ mice.^20^ Notably, compared to mice that received an equal but continuous load of high-fat diet, immune training through intermittent diet showed more advanced atherosclerotic progression.^20^ This was found to be rooted in metabolic and epigenetic reprogramming of neutrophils to a pro-inflammatory state, suggesting maladaptation through central trained immunity.^20^ Notably, neutrophil phenotype and abundance were linked to transcriptional downregulation of *Runx1* in GMPs.^20^ The role of RUNX1, a transcription factor regulating neutrophil cytokine responses at the level of GMPs, was confirmed through a lineage specific *Runx1* knock-out.^20^ In an earlier study, a high-fat diet was demonstrated to trigger epigenetic and pro-inflammatory alterations in the bone marrow of mice.^119^ Bone marrow transplantation from *Ldlr*^*−/−*^ mice fed 45 weeks of high-fat diet caused macrophage-driven progression of atherosclerosis in naïve *Ldlr*^*−/−*^ recipient mice. High-fat-diet-induced epigenetic reprogramming in the bone marrow was marked by decreased DNA methylation of gene loci encoding PU.1 and IRF8, transcription factors involved in myeloid differentiation.^119^

Similar indications of trained immunity induction were found in models of hyperglycemia. Trained immunity phenotypes in response to hyperglycemia can be observed in mouse macrophages and human monocytes in vitro.^120^ In a bone marrow transplantation study from diabetic mice into *Ldlr*^*−/−*^ recipient mice, hyperglycemia-induced central trained immunity was identified as a driving factor of atherosclerosis.^17^ In this context, a role of RUNX1 in hyperglycemia-induced trained immunity was indicated by motif analysis of differentially accessible chromatin in hematopoietic progenitors.^17^ Pharmacological inhibition of RUNX1 was found to diminish hyperglycemia-induced trained immunity phenotypes in vitro.^17^ Importantly, this role of RUNX1 contrasts the findings of its role in high-fat diet studies.^17,20^ This suggests that maladaptive trained immunity is more complex than one homogeneous inflammatory program. Together, these findings demonstrate how inflammation induced by lifestyle factors can amplify the atherοsclerotic process through induction of central trained immunity.

**Fig. 3:**
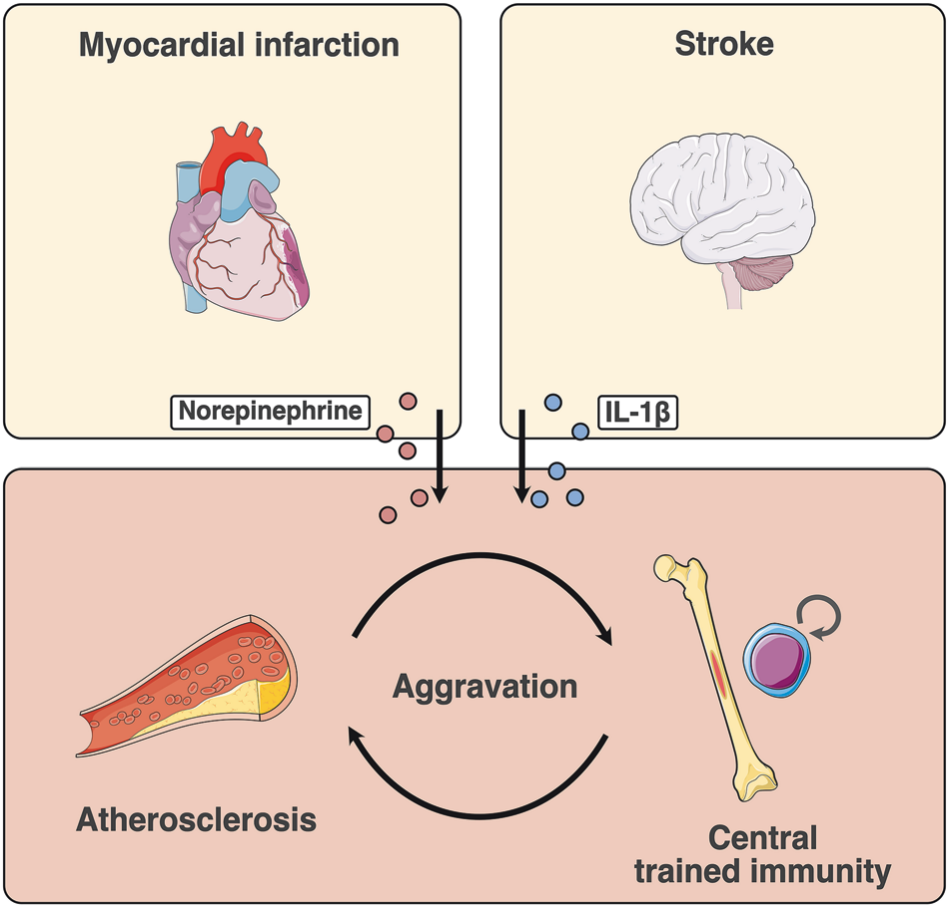
Central trained immunity aggravates atherosclerosis. Atherosclerosis and diet associated inflammation can reprogram bone marrow derived-innate immune cells. This maladaptive trained immunity can promote aggravation of atherosclerosis. Severe atherosclerosis possibly leads to acute complications such as myocardial infarction or stroke. These severe acute incidences can lead to a second induction of maladaptive trained immunity with norepinephrine and IL-1β identified as key mediators identified in myocardial infarction and stroke, respectively.^19,22^ If not counteracted, this interplay can result in a spiral of disease aggravation.

In certain circumstances, the atherosclerotic plaques may reach a critical mass or level of instability resulting in their rupture. Such acute events can lead to complications such as myocardial infarction or stroke, each with distinct inflammatory phenotypes. Previous work revealed that the systemic inflammation that occurs after myocardial infarction increases atherosclerotic progression and affects regulation of the bone marrow.^121,122^ Bone marrow transplantation experiments identified maladaptive central trained immunity in an experimental mouse model of myocardial infarction, driving atherosclerotic progression in naïve recipient mice.^19^ Maladaptive trained immunity in the bone marrow was linked to sympathetic nerve activation and norepinephrine release.^19^ Furthermore, myocardial infarction may have strong implications for the metabolism in the bone marrow niche. Bone marrow adipocytes were shown to fuel emergency hematopoiesis after myocardial infarction.^123^ Sympathetic nerves mediate the release of fatty acids from local adipocytes and thereby influence the metabolism of HSPCs, possibly affecting trained immunity.^123^ In a different model, sympathetic denervation leading to decreased TGF-β release in the bone marrow was identified to underlie maladaptive trained immunity in the context of heart failure.^21^ These findings suggest a prominent role of the sympathetic nervous system in regulating bone marrow HSPCs and central trained immunity (Fig. 3).

Stroke is another primary acute complication from plaque rupture, leading to ischemic brain injury. Initially, a systemic pro-inflammatory response is triggered by the release of DAMPs, which then shifts to an immunosuppressed state.^124^ After immunosuppression follows a chronic pro-inflammatory state characterized by circulating DAMPs and inflammatory cytokines. Recent studies suggest a trained immunity component associated with stroke.^22,125,126^ In a mouse model of stroke, systemic release of IL-1β causes maladaptive trained immunity in bone marrow HSPCs, identified with bone marrow transplantation.^22^ Pro-inflammatory monocytes were found to infiltrate the myocardium contributing to cardiac dysfunction through fibrosis. Notably this was observed without secondary challenge of naive recipient mice.^22^ These experiments suggest implications of stroke far beyond the brain and demonstrate the interplay between brain and heart in the context of trained immunity and ASCVD (Fig. 3).

The multifactorial nature of ASCVD complicates the identification and design of effective therapeutic interventions.^127^ Treatment of atherosclerotic progression may benefit from lifestyle interventions that specifically avoid diets associated with maladaptive trained immunity. Similarly, if a patient has suffered a myocardial infarction or a stroke, therapeutic intervention may be necessary to counteract maladaptive trained immunity.

### Cancer

Cancer progression and metastasis are influenced by local factors and systemic changes driven by the tumor.^128,129^ The tumor microenvironment (TME) can release various mediators, especially metabolites, to modulate different functions of the body.^130–132^ A key example are the immune responses that are remodeled by the tumor. Cells like tumor-associated macrophages (TAMs), neutrophils (TANs), and MDSCs are well-studied for their pro-tumor biological activity.^133^

Several mouse studies demonstrated tumor-induced changes in hematopoietic organs.^134,135^ For instance, continuous secretion of G-CSF in mice bearing large tumors leads to a profound regulatory shift in the bone marrow and spleen, linked with expansion of HSPCs.^128^ While intermittent G-CSF is necessary for granulocyte maturation, continuous exposure to tumor-derived G-CSF was shown to steer granulopoiesis toward T cell-suppressive neutrophils.^136^ Similarly, tumor-induced IL-3 overexpression increases common myeloid progenitors (CMPs), GMPs, and megakaryocyte-erythrocyte progenitors (MEPs) in both bone marrow and spleen.^137^

In clinical studies, blood samples from cancer patients at various stages contained myeloid cells lacking maturation markers. These immature myeloid cells stem from the bone marrow and actively suppress antigen-specific T cell responses.^138^ Single cell transcriptomic analysis of circulating myeloid cells of thyroid carcinoma patients indicated a decreased inflammatory capacity.^139^ These cells display lower cytokine production capabilities and overproduced ROS.^139^ Similar signatures were found in HSPCs, highlighting the tumors’ ability to pre-program these cells before they enter the TME.^139^

Megakaryocytes are also significantly affected by the TME. IL-6 produced by tumors up-regulates hepatic thrombopoietin, leading to thrombocytosis due to increased platelet production by megakaryocytes.^140^ TME regulation of megakaryopoiesis appears to affect the phenotype of the platelets. Megakaryocytes from tumor-bearing mice were found to exhibit a pro-inflammatory phenotype displaying increased *Ctsg*, *Lcn2*, *S100a8*, and *S100a9* transcripts.^141^ The functional reprogramming was partly transferred to platelets, equipping them with pro-inflammatory proteins that promote tumor invasion and metastasis.^141^ Another study showed that hyperglycemia, an established risk factor for tumor metastasis, was shown to alter megakaryocyte metabolism, leading to the production of platelets with increased in vitro adherence to melanoma cells.^142^

**Fig. 4:**
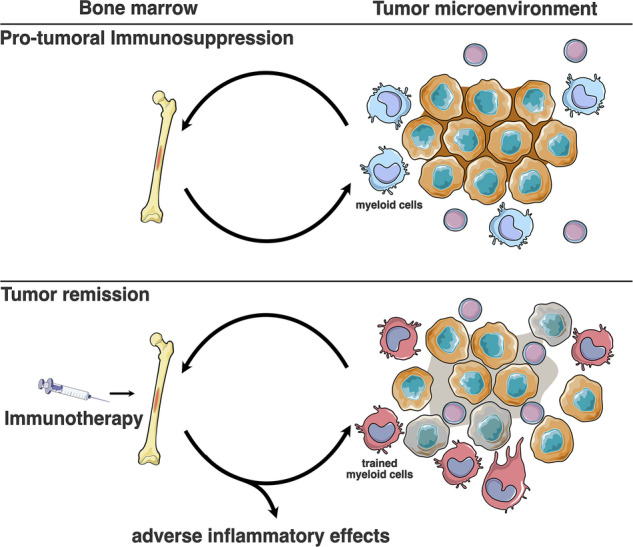
Induction of central trained immunity as cancer immunotherapy. Factors released by the tumor, such as G-CSF and IL-3 induce changes in the hematopoietic system leading to a myeloid cell compartment that can promote tumor growth and metastasis and suppress eventual adaptive immune responses. Immunotherapy aimed at inducing trained immunity in the hematopoietic system can revert pro-tumoral immunosuppression and promote a pro-inflammatory phenotype in tumor associated myeloid cells, eventually supporting adaptive responses and leading to remission.

These findings suggest that targeting hematopoietic organs could counteract the systemic immunosuppression driven by the TME. One approach involves inducing trained immunity (Fig. 4). Trained immunity inducers BCG and β-glucan are extensively studied for displaying promising anti-tumor effects through trained immunity, as reviewed elsewhere.^143,144^ For instance, intraperitoneal injection of mice with β-glucan diminished tumor growth upon inoculation with B16F10 cells 7 days or even 28 days later.^145^ This effect was found to be driven by the anti-tumor phenotype of neutrophils present in the TME.^145^

Similar results were observed using a muramyl tripeptide-loaded nanoparticle (MTP-HDL) targeting the bone marrow.^146^ Mice treated with MTP-HDL after B16F10 cell inoculation displayed significant reduction in tumor growth.^146^ Most interestingly, when given in combination with checkpoint inhibitor therapy, MTP-HDL had a potentiating effect in this otherwise refractory model for checkpoint inhibitory therapy.^146^ Both these studies employed bone marrow transplantation to prove that the observed effects are, at least in part, long lasting and mediated by central trained immunity.^146^ Similarly, peripheral trained immunity antitumor phenotypes can be induced in alveolar macrophages upon acute respiratory viral infection.^40^ Trained alveolar macrophages were resistant to tumor-associated immune suppression during lung tumor development.^40^

HSC-targeting gene therapy is another potential approach to treat solid tumors. This was demonstrated using a lentiviral expression system to induce expression of a dominant-negative mutant of TGF-β receptor II driven by the heat inducible heat shock protein 70B promoter.^147^ Upon bone marrow transplantation from transfected mice, naïve recipient mice displayed significant reduction in tumor growth after GL261 tumor cell inoculation 3 weeks after the transplant.^147^ These studies show that targeting the bone marrow is an effective and novel way of treating cancer that undercuts the systemic immunosuppressive program induced by the TME on immune cells.

Despite advances in immunotherapy, surgical resection, irradiation and cytotoxic chemotherapy remain the foundational treatment for most cancers to date. Through removal of secondary lymphoid organs and cytotoxic effects on immune cells, these therapies have a strong impact on immune regulation. This can support immunogenicity by triggering immunogenic forms of tumor cell death and increasing dispersion and presentation of tumor antigen.^148^ While there are indications of peripheral trained immunity through glioblastoma radiotherapy in microglia, it is unclear how central trained immunity is affected.^149^ Induction of central trained immunity with β-glucan is reported to sustainably enhance myelopoiesis and could be employed to counteract ablative effects on the hematopoietic system caused by chemotherapy. This was demonstrated in mouse models of chemotherapy-induced myeloablation.^14^

These studies suggest that trained immunity effects can not only be considered for novel therapy, but also in combination with established treatments.

### Neurodegenerative disease

The CNS is an immune-privileged organ.^150^ Microglia are self-renewing yolk-sac-derived phagocytes and the most prevalent representatives of innate immunity in the CNS. Microglia maintain homeostasis by phagocytosis of protein aggregates and other cellular debris and respond to PAMPs and DAMPs, initiating neuroinflammation.^151,152^ Alzheimer’s disease is the most prevalent neurodegenerative disease characterized by aggregation of endogenous amyloid-β protein extracellularly, and tau protein derived neurofibrillary tangles intracellularly.^153^ The second most prevalent neurodegenerative disease after Alzheimer’s disease is Parkinson’s disease, marked by intracellular fibrillar aggregation of α-synuclein protein in dopaminergic neurons.^153^ Neuroinflammation is regarded as a driving factor of both Alzheimer’s and Parkinson’s disease, with an important axis being microglial NLRP3 inflammasome activation and IL-1β release associated with exposure to pathological protein aggregates.^151,154,155^ Single-cell transcriptomics of a murine Alzheimer’s disease model uncovered a cell state of disease-associated microglia only found at sites of neurodegeneration.^156^ Long-term immune reprogramming is known in microglia and termed microglial priming, which shares similarities with trained immunity and is known to play a role in neurodegenerative disease.^96^ Understanding the role of immune regulation and innate immune memory in microglia and their apparent subsets is of high therapeutic interest.

Risk factors for Alzheimer’s disease overlap with known factors causing maladaptive trained immunity, including brain trauma, periodontitis, obesity and systemic inflammation and infection.^16,18,22,151,157^ Similar to Alzheimer’s disease associated neuroinflammation, maladaptive central trained immunity associated with periodontitis, brain injury and western-like diet induced inflammation is induced by inflammasome signaling and IL-1β.^16,18,22^ Contributions of central and peripheral maladaptive trained immunity linking periodontitis and diet with AD remain to be studied.

Under homeostasis, microglia are separated from monocyte-derived macrophages through the blood-brain barrier, but neuroinflammation can allow monocyte infiltration into the CNS.^158^ Elevated circulation high-mobility group box 1 (HMGB1), a DAMP capable of inducing trained immunity, is a biomarker for Alzheimer’s disease associated neuroinflammation and blood-brain barrier dysfunction.^159,160^ Protein aggregates associated with Alzheimer’s and Parkinson’s disease are also recognized as DAMPs through multiple PRRs. Subsequent activation of innate immune cells can promote clearance of protein aggregates or exaggerate disease through excessive neuroinflammation.^152^ Whether these processes can lead to long-term maladaptive trained immunity remains to be explored.

When injected intraperitoneally, both β-glucan and LPS induce trained immunity in microglia.^161^ A single dose of LPS induces tolerance systemically but induces trained immunity in microglia.^157^ Alzheimer’s disease-prone APP23 mice challenged once with LPS display increased amyloid-β plaque formation and decreased release of immunoregulatory IL-10 in the brain.^157^

While acute brain injury and the associated NLRP3 inflammasome/IL-1β signaling is described to induce central maladaptive trained immunity, a similar phenotype is observed peripherally in microglia.^22,126^ Microglial training was reported to be induced by brain injury caused by glioblastoma radiotherapy.^149^ Microglia also displayed long-term maladaptive trained immunity in a mouse model of cortical microinfarct, a co-morbidity of Alzheimer’s disease and stroke.^126,162,163^ Maladaptive trained immunity exacerbated inflammation and injury in a photothrombotic mouse model of stroke four weeks later.^126^ Independent of acute inflammasome signaling, NLRP3 was found to enter the nucleus and interact with histone methyltransferase mixed-lineage leukemia 1 (MLL1) to induce maladaptive epigenetic memory, which can be blocked by eliminating microglial NLRP3 in inducible knock-out models.^126^

**Fig. 5:**
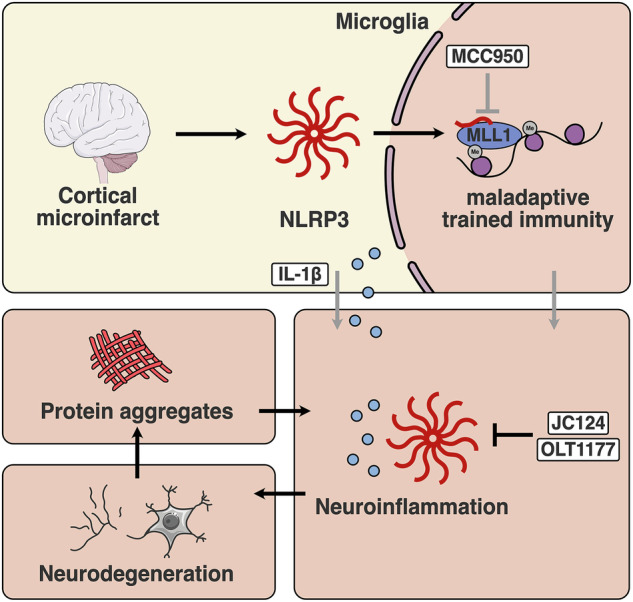
NLRP3 activation in neurodegenerative disease as a potential factor of reprogramming. Brain injury can activate the NLRP3 inflammasome in microglia, leading to IL-1β-mediated inflammation. In the same context, NLRP3 enters the nucleus and interacts with MLL1 leading to maladaptive trained immunity.^126^ Microglial NLRP3- and IL-1β-mediated neuroinflammation in response to pathologic protein aggregates also plays an important role in the progression of neurodegenerative disease.^152^ Whether NLRP3 activation can lead to trained immunity in microglia in the context of neurodegenerative disease is yet unclear. Inhibition of NLRP3 activation with JC124 and OLT1177 can ameliorate neurodegeneration in mouse models.^166,168^ MCC950 was shown to affect NLRP3 functions unrelated to classical activation in mouse models of Alzheimer’s disease.^165^ It is yet unclear whether these inhibitors can affect the role of NLRP3 in trained immunity.

Genetic ablation of NLRP3 in Alzheimer’s disease-prone APP-PS1 mice prevents disease progression.^164^ In the same knock-out model, metabolic and epigenetic alterations associated with glutaminolysis and phagocytotic uptake of amyloid-β can be observed in microglia.^165^ Τhis effect could be recapitulated through chronic administration of a small molecule NLRP3 inhibitor ex vivo. NLRP3 inhibitors OLT-1177 and JC-124 were found effective against disease progression in APP-PS1 mice with OLT-1177 also showing efficacy in a Parkinson’s disease mouse model.^155,166–168^ Given the efficacy of canakinumab and anakinra as blockers of the downstream signaling component IL-1β, it can be hypothesized that NLRP3 blockade is a promising upstream target. NLRP3 inhibition would block both IL-1β release and pyroptotic cell death with the associated release of other DAMPs, while maintaining full function of other inflammasome sensors.^169^ While for many candidates only preclinical evidence is available, effective doses of OLT-1177 were found safe in humans.^170^ The effects of NLRP3 inhibitors on its reported functions in epigenetic and metabolic regulation and maladaptive trained immunity in microglia remain to be investigated^126,165^ (Fig. 5).

## Conclusion

Research performed in the last decade has demonstrated long-term changes in innate immune responses upon challenge with various exogenous and endogenous ligands, a de-facto memory characteristic of innate immunity termed trained immunity. Long-term epigenetic and functional reprogramming of both peripheral innate immune cells and their bone marrow progenitors represent the basic mechanisms of trained immunity. While these processes were unknown for a long time, we have used vaccines and adjuvants that reprogram our hematopoietic progenitors and peripheral innate immune cells for over a century, with a massive impact on public health through their heterologous protection against pathogens and reductions in all-cause mortality. While inducing trained immunity is protective against infection and other conditions characterized by immunosuppression such as cancer, inappropriate induction by endogenous ligands can lead to long-term sterile inflammation and disease. This maladaptive trained immunity appears highly context-dependent with diverse mechanisms involved. We are in the beginning of understanding these processes and the molecular mechanisms that underlie them. Understanding these is a crucial step towards the development of novel therapeutic approaches for the prophylaxis and treatment of diseases in which trained immunity plays a central role.
